# Patagonian partnerships: the extinct *Dusicyon avus* and its interaction with prehistoric human communities

**DOI:** 10.1098/rsos.231835

**Published:** 2024-04-10

**Authors:** Cinthia C. Abbona, Ophélie Lebrasseur, Francisco J. Prevosti, Eva Peralta, Lucio González Venanzi, Laurent Frantz, Greger Larson, Adolfo F. Gil, Gustavo A. Neme

**Affiliations:** ^1^ Instituto de Evolución, Ecología Histórica y Ambiente (IDEVEA), UTN-CONICET, Avenue Gral. Urquiza 314, CP5600, San Rafael, Mendoza, Argentina; ^2^ Palaeogenomics and Bio-Archaeology Research Network, School of Archaeology, University of Oxford, Oxford OX1 3TG, UK; ^3^ Museo de Ciencias Antropológicas y Naturales, Universidad Nacional de La Rioja (UNLaR), Avenue Luis M. de la Fuente S/N, La Rioja 5300, Argentina; ^4^ Div. Arqueología, Anexo Museo, Laboratory 128 (FCNyM-UNLP), La Plata, Argentina, FHumyAr (UNR), Rosario, Argentina; ^5^ Graduate School Life Science Munich, Faculty of Biology/Biocenter, Grosshadernerstr, 2-4, 82152 Planegg-Martinsried, Munich, Germany

**Keywords:** South American canid, *Dusicyon avus*, ancient DNA, stable isotope analysis (δ^13^C_col_, δ^15^N), extinct fox

## Abstract

The southern Mendoza province, located in the northern region of Patagonia, was inhabited by hunter-gatherer groups until historic times. Previous archaeological studies have reported canid remains among faunal assemblages, which were assumed to be part of the human diet. However, the taxonomic identification and significance of these canids within human groups have raised questions. In this study, we used ancient DNA analysis, morphological examination and stable isotope analysis (δ^13^C_col_ and δ^15^N) to re-evaluate the taxonomic assignment of a canid discovered at the Late Holocene burial site of Cañada Seca. Previous morphological identifications suggested that it belonged to the genus *Lycalopex*, but our results conclusively demonstrate that the individual belongs to the extinct fox species *Dusicyon avus*. This finding expands *Dusicyon avus’* known geographical distribution to Patagonia’s northern extremity. Furthermore, statistical predictions based on genetic divergence undermine the hypothesis that hybridization between *Canis* and *Dusicyon*, facilitated by the introduction of domestic dogs, played a role in the extinction of *Dusicyon* species. On the other hand, our findings indicate that a *Dusicyon avus* individual shared a similar diet and was probably buried alongside humans, suggesting a close relationship between the two species during their lives and deaths.

## 1. Introduction

South America is home to a large diversity of canids that encompasses 11 extant species: *Atelocynus microtis*, *Cerdocyon thous*, *Chrysocyon brachyurus*, *Speothos venaticus*, *Urocyon cinereoargenteus* and six species belonging to the genus *Lycalopex* [1–4]. Additionally, two now-extinct canid species once inhabited this area: *Dusicyon avus* and *Dusicyon australis*, the latter of which is commonly known as the Falkland/Malvinas Islands’ wolf and is exclusively found on these islands [1,5]. Palaeogenetic studies have shown the divergence between these two *Dusicyon* species occurred approximately 16 000 years BP [6].

*Dusicyon avus* is a medium-sized canid with an estimated body mass of 10–15 kg [7]. There have been instances of *D. avus* specimens overlapping in size with *L. culpaeus* [5,7]. The fossil record of *D. avus* primarily consists of skulls, jaws and teeth, spanning the Late Pleistocene (Lujanian, 126 000–*ca* 10 000 years BP) until its extinction approximately 500 years BP [5]. Its geographical distribution was extensive, encompassing southern Brazil, Uruguay, northeast Argentina, Pampa and Patagonia [5,8–11]. The species inhabited various open areas, including shrub and herbaceous steppes, exhibiting a range of climatic conditions [5].

The recent extinction of *D. avus* is probably attributable to the reduction in its geographical distribution caused by climate change and increased anthropogenic impact [5,7]. The introduction of domestic dogs in South America from Mesoamerica by indigenous people *ca* 5000 years BP [12] and their arrival in Patagonia *ca* 700–900 years BP [13,14] may have also led to hybridization events between the two species, potentially further contributing to its extirpation [8]. Yet, the possibility of such hybridization remains to be confirmed. Furthermore, genetic studies focusing on the species within the *Dusicyon* genus have been scarce, yet understanding these is needed to comprehend the evolutionary history of *D. avus* and *D. australis*, their historical distributions and the natural and anthropogenic factors that would have contributed to their demise.

### 1.1. Roles of canids in South American human societies

Ethnographic research has demonstrated the different types of relationships between wild animals and humans that can arise from their interactions, from that of prey to that of pet [15,16]. For instance, in Amazonian indigenous communities, young wild canid species are commonly adopted, become part of the family and are treated like humans when they die [16–18]. This interaction is perhaps best illustrated by the social, symbolic and economic nature of our relationships with the domestic dog (*Canis familiaris*), which has not only been a hunting aid, guard and companion but has also been part of medicinal and symbolic practices, a source of meat and fur and a hauling animal, among others roles [19–24]. Beyond human’s best friend, societies have equally maintained diverse relationships with wild canid species sharing their environment [25–29]. Additionally, ancient dogs’ remains have also been extensively studied because of their potential as proxies for human palaeodiet [30–35].

Conversely, research on the relationships between people and wild canids, particularly foxes, has been limited [26–28,36]. Foxes probably held significant economic and symbolic roles within South American societies, as indicated by their frequent presence on archaeological sites [5,7,29,37,38]. For example, teeth belonging to canid species such as *Lycalopex sechurae*, *L. culpaeus*, *Lycalopex gymnocercus* and *Ch. brachyurus* were used as personal ornaments and were found in human burials from Argentina and Peru [25,39,40]. Among these species, *L. culpaeus* was involved in ritual activities in the highlands of Bolivia, such as accompanying buried human individuals and building offerings [38,40]. In other archaeological sites in Chile and Argentina, the remains of these species have been interpreted as occasionally part of the human diet [41,42].

Among the various wild canid species, *D. avus* shows the most robust evidence of interaction with prehistoric societies in the Southern Cone. Numerous archaeological records of this extinct fox are associated with human occupations [5,37,42–45], suggesting significant and distinctive symbolic connections between both species [4,7,37,43,44,46]. For instance, at the Loma de los Muertos site, an individual was buried individually in a mortuary area and interpreted as a pet [44]. At the Río Luján 1 site, a skull of this species was placed as a grave good for a human burial [47]. Additionally, their canines may have been used as personal ornaments [48]. Despite this strong relationship with the symbolic sphere, zooarchaeological studies indicate that *D. avus* was not usually eaten by humans [5,7,37,44], aligning with the consumption patterns of other canid species in the Southern Cone.

**Figure 1.**
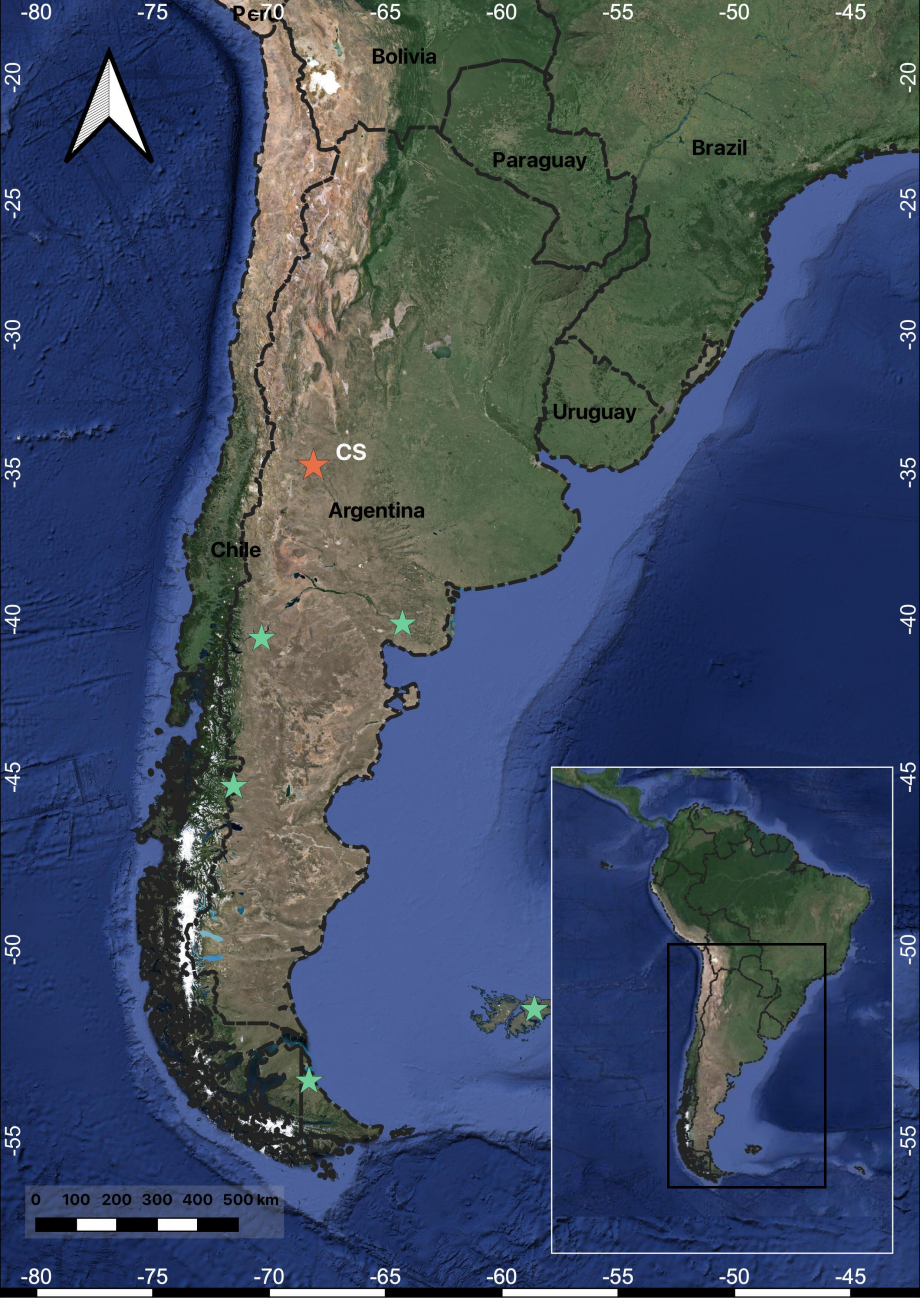
Map of the Southern Cone of South America. The red star indicates the location of the Cañada Seca (CS) archaeological site, while the green stars represent other sites with available *Dusicyon* sequences on GenBank.

In 1991, an archaeological rescue excavation was conducted at the pre-Hispanic burial site of Cañada Seca, Argentina, primarily inhabited by hunter-gatherer communities (figure 1). In addition to human osteological remains, canid bones belonging to an almost complete individual were discovered and initially identified as belonging to the *Lycalopex* genus based on morphological characteristics [49]. In the present work, we look to study in-depth the bone remains of this canid; the objectives of this study are: (i) to confirm the species identification and chronological age of this canid through a comprehensive analysis of morphological characteristics, ancient DNA and radiocarbon dating; (ii) to assess the specific interactions, such as synanthropy and shared feeding practices, between humans and the buried canid through palaeodiet analysis; and (iii) to investigate the potential role of hybridization between *Dusicyon avus* and dogs as a contributing factor to extinction. We hypothesize that confirming the species identification and chronological age of the discovered canid, along with characterizing its diet and exploring its potential reproductive compatibility with domestic dogs, will shed light on the ecological dynamics and human–animal interactions within the hunter-gatherer.

### 1.2. Cañada Seca archaeological site

The Cañada Seca archaeological site is located in the middle valley of the Atuel River (−34.7506944 S, −69.02175 W), at 596 m a.s.l., in northwestern Patagonia (figure 1). After its accidental discovery owing to clay mining activities, the Museo de Historia Natural de San Rafael conducted a rescue excavation at the site to prevent further looting and disturbance [49,50]. Unfortunately, much of the contextual information was lost owing to the nature of the excavation, hindering a detailed understanding of the site’s formation process and stratigraphic context as a mixed multiple burial site. Nevertheless, archaeological analyses of the recovered material [49,51] have provided valuable contextual information about Cañada Seca and its buried individuals.

A total of 3470 human bones were recovered from the site, representing a minimum of 24 individuals, including three infants, one child, two adolescents and 18 adults. The non-human remains found at the site belonged exclusively to canids, except for small mammals (e.g. rodents) that have a non-anthropogenic origin [49]. Grave goods discovered at the site include personal ornaments such as necklace beads, lip ornaments, tembetás (lip ornaments) and stone tools such as projectile points.

The site has been radiocarbon dated to approximately 1500 calibrated years BP (with a median date of 1398 cal BP and sigma range between 1314 and 1514 years BP) based on five radiocarbon dates obtained from human bones [49,52,53]. This community was part of a larger population that inhabited the region with a non-sedentary settlement pattern. The δ^18^O isotope analysis suggests that individuals at the site had high mobility though within a range not exceeding 70 km. Furthermore, the δ^13^C_col_ and δ^15^N values indicate a low-level food production within the community, with domestic plants like *Zea mays* comprising less than 10% of their diet [49].

## 2. Material and methods

### 2.1. Taxonomic identification

The Cañada Seca faunal assemblage comprised 496 bones for which the zooarchaeological analysis can be found in Peralta *et al*. [49]. The CS/91 canid specimen is preserved as a partially mounted skeleton, plus several separated postcranial bones, incomplete mandibles and a skull (see the electronic supplementary material, tables S1 and S2). These bones agree in their colour and preservation, size and morphology and justify the interpretation that they all belong to the same individual.

**Figure 2.**
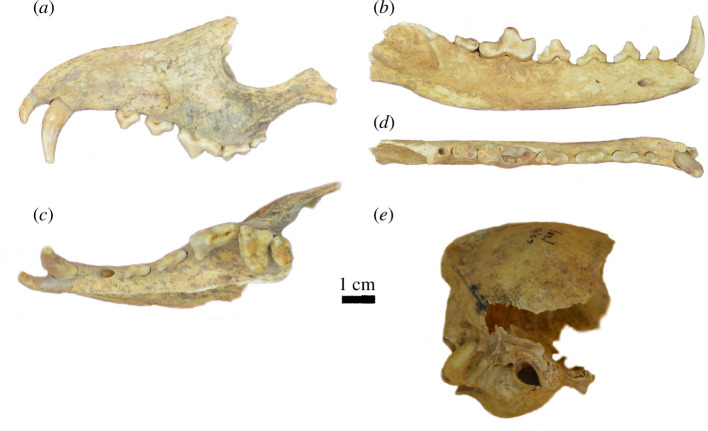
Cranial and mandible remains of the CS/91. (*a*) Maxilla in lateral view. (*b*) Maxilla in ventral view. (*c*) Right mandible in lateral view. (*d*) Right mandible right in dorsal view. (*e*) Braincase in right lateral view.

The canid from Cañada Seca (figure 2; electronic supplementary material, figure S1) was morphologically compared (electronic supplementary material, figures S2 and S3) with canids from the Southern Cone of South America (*Lycalopex* spp., *Ce. thous*, *D. avus* and *Ca. familiaris*), following criteria and characters discussed in [5,7,14,21,54]. Extinct large canids (e.g. *Aenocyon dirus*) and living *Ch. brachyurus* were excluded from the comparison owing to significantly larger sizes and different anatomical patterns [54].

Owing to more diagnostic features in the skull and dentition and the lack of published descriptions of the postcranial skeleton of extinct taxa (i.e. *Dusicyon* spp.), we have focused our comparison specifically on the skull and dental characters. Measurements were taken using a digital calliper with a precision of 0.01 mm (see the electronic supplementary material, table S2: Measurement section-mm). Bivariate graphs and principal component analyses (PCA) were based on the variance/covariance matrix of the log-transformed measurements (electronic supplementary material, figures S20–S22; tables S3–S5).

We test the hypothesis that the CS 91/1 specimen belongs to *D. avus* by calculating the coefficient variation (CV; e.g. [55]), and comparing it with the values obtained in a living sample of *L*. *culpaeus* [5] (see variation coefficients in the electronic supplementary material, table S2). In the cases in which the CV of CS 91/1 is larger than the CV of *L*. *culpaeus*, the significance of the difference was evaluated with the Cope and Lacy and the Fligner–Killeen tests following Plavcan & Cope [55] (electronic supplementary material, table S2). The Fligner–Killeen test was evaluated with 1000 resamplings, while the Cope and Lacy test was tested with 100 000 permutations [55]. All the statistical analyses were performed in R, version 4.0.2 [56] through RStudio, version 1.3.1073 [57].

### 2.2. DNA extraction and sequencing

For ancient DNA analyses, two canid bones were chosen: a left radius (CS/91-1; electronic supplementary material, figure S23) and a caudal vertebra (CS/91-2). The extraction and library building were conducted in a dedicated ancient DNA laboratory at the Palaeo-BARN, School of Archaeology, University of Oxford, UK. To minimize the risk of environmental contamination, the bone surface was carefully prepared by removing 0.5–1 mm using a Dremel 3000 electric hand drill. Samples weighing 100 and 90 mg were taken from CS/91-1 and CS/91-2, respectively, and ground into a fine powder using a Retsch MM400 microdismembrator. DNA extractions followed the method described previously [58] with modifications based on [59], including a 30 min pre-digestion step. Subsequently, double-strand libraries were constructed according to the protocol outlined [60]. The libraries were sequenced on an Illumina HiSeq2500 platform at the Centre for GeoGenetics, Copenhagen, using 80 bp single-end sequencing, resulting in 868 131 raw sequencing reads. Adapter sequences were trimmed from each sequence to ensure high-quality data using SeqPrep [61]. Finally, ancient DNA degradation was analysed using the mapDamage tool [62], which involves modelling post-mortem DNA damage based on patterns of nucleotide misincorporations for each library.

### 2.3. Data processing

The current availability of whole mitochondrial genomes for South American wild canid taxa is extremely limited, with only the maned wolf (*Ch. brachyurus*, Genbank Accession NC_024172) and the sechuran fox (*L. sechurae*, Genbank Accession KT448284) mitochondrial genome available. To overcome this limitation, we mapped our reads to the complete mitochondrial genomes of the domestic dog (*Ca. familiaris*), the maned wolf and the sechuran fox (Genbank Accession NC_002008) using the iterative assembler MIA [63]. Additionally, we included a second domestic dog specimen from Argentina dating to the late Holocene as a positive control (Genbank Accession KF661084.1; electronic supplementary material, table S6). A position-specific substitution matrix for damaged ancient DNA was implemented to enhance alignment accuracy and consensus calling. Bases with 3× coverage and agreements exceeding 66% were selected. Mitochondrial nucleotide positions without sequence data were labelled as ‘N’ for missing data. The consensus sequence was aligned against each reference genome using ClustalW and Geneious 7.1.9 [64]. Owing to the lack of mitochondrial reads and poor DNA preservation of CS/91-2, no further analyses were conducted on this bone. The public Sequence Read Archive (SRA) [65] submission for CS/91-1 is PRJNA1001383.

### 2.4. Sequence alignment

Given the limited availability of South American canid species based on whole mitochondrial genomes, we focused on targeting specific fragments of the cytochrome *c* oxidase subunit II (COII) and cytochrome *b* genes (cytb), which allowed us to include a broader dataset of species. Therefore, we generated and aligned the sequences of these two fragments from CS/91-1 with the reference sequences of *Ca. familiaris* and with available sequences of South Am erican wild canids. The dataset included 10 *Dusicyon* specimens [6] and eight South American canids previously studied [66]: *A. microtis*, *Ce. thous*, *Ch. brachyurus*, *L. culpaeus*, *L. gymnocercus*, *L. sechurae*, *Lycalopex griseus* and *S. venaticus*. The GenBank accession numbers for these sequences can be found in the electronic supplementary material, table S7.

### 2.5. Phylogenetic analyses

Previous studies have demonstrated the monophyly of South American canids (e.g. [6,66]). To determine the phylogenetic placement of our CS/91-1 specimen, we conducted Bayesian and maximum likelihood (ML) phylogenetic analyses using the COII and cytb datasets (electronic supplementary material, table S7). For Bayesian analysis, we used BEAST v.1.10.1 [67]. The substitution model selection was based on comparing different models using MODELTEST 3.7 [68] and following Akaike information criterion scores. The HKY+G model was identified as the best fit for the COII data, while the HKY+I+G model was optimal for the cytb dataset. The Markov chain Monte Carlo was run for 50 000 000 iterations, with trees sampled every 5000 iterations and a burn-in of 5 000 000 states. ML analyses were performed using Garli 0.951 [69] under the HKY+G+I substitution model, with 1000 bootstrap (BS) replicates.

### 2.6. Reproductive compatibility

Previous research has shown that mitochondrial genetic divergence between pairs of terrestrial mammalian species can be used as a reliable indicator to predict relative hybrid sterility in their offspring [70]. To examine whether mating between dogs and *Dusicyon* species could result in fertile hybrid offspring, we calculated the average pairwise genetic distance of the cytb gene between the following species pairs: *Ca. familiaris* × *D. avus* and *Ca. familiaris* × *D. australis*. This analysis was performed using Geneious 7.1.9 [64]. Owing to the absence of complete cytb sequences in the *Dusicyon* samples, we focused on a 283 bp fragment of the cytb gene for observation. We compared these genetic distance values to the two categories established by Allen *et al.* [70]: category 1, which includes mammalian species pairs capable of producing fertile hybrid offspring without backcrossing, and category 2, which includes mammalian species pairs producing infertile hybrid offspring or offspring that follow Haldane’s rule. Additionally, we calculated these values to assess the extent of hybrid sterility between *D. avus* and *D. australis*.

### 2.7. Stable isotope analysis and radiocarbon dating

The CS/91-1 radius fragment was also analysed for stable isotopes according to the bone collagen extraction protocol of the Laboratorio de Isótopos Estables en Ciencias Ambientales-LIECA [71]. Stable carbon and nitrogen isotopic compositions were determined using a Thermo Scientific DELTA V advantage continuous flow isotope ratio mass spectrometer coupled via ConFlo IV to an Elemental Analyzer Flash 2000 at LIECA (CONICET & UTN FRSR; electronic supplementary material, table S8). Radiocarbon Accelerator mass spectrometry (AMS) measurements were conducted at Penn State’s Radiocarbon Laboratory. The obtained data (electronic supplementary material, table S8) was calibrated 2σ using the OxCal v.4.4.4 program and the southern hemisphere curve (SHCal20) [72,73]. The isotopic values obtained for the canid were compared with other carnivores from the region ([74]; corrected for the Suess effect) and with the human bones found at the Cañada Seca site (details in [49]).

## 3. Results

### 3.1. Morphological species identification

The specimen from Cañada Seca (CS/91; figure 2; electronic supplementary material, figures S1–S3) is nearly complete, except for a broken skull that retains certain parts: an incomplete braincase (figure 2*e*); a fragment containing part of the left premaxilla, maxilla and zygomatic bone with teeth I3, C1, P2–M2 (figure 2*a,b*); a right zygomatic bone (electronic supplementary material, figure S1*f*); an incomplete right mandible with teeth c1–m2 (figure 2*c,d*); and an incomplete left mandible with teeth c1, p2–p3, a broken m1 and the m3 (electronic supplementary material, figure S1*a*). The dentition has advanced wear on the carnassial and main cusps of other teeth. The left m1 is extremely worn, with the pulp chamber exposed, and the talonid appears to be broken during the specimen’s lifetime, something that probably is related to the presence of an exostosis around the m2–m3 alveolus, and especially on the lateral wall of the mandible below the m1 talonid and m2–m3 (electronic supplementary material, figure S1*b*).

The overall size of the specimen is comparable to that of a large fox, such as *L. culpaeus* and *Dusicyon* spp. (electronic supplementary material, table S2). The superficial masseteric scar on the zygomatic bone is as wide as in the canids of the ‘South American clade’ [75–77] and different from *Canis*. The braincase is proportionally wider than *L. culpaeus* and more similar to *Dusicyon* spp. The bullas are not as depressed as in *Ca. familiaris* and are proportionally more inflated than in *L. culpaeus*, a feature shared with *Dusicyon* spp. The oval foramen is more rounded and less elliptical than in *L. culpaeus*.

The morphological pattern of the dentition also agrees with a large South American fox and differs from *Ca. familiaris*. The canines and premolars are more gracile, the principal cusps of the premolars are more acute and the paracone of the M1 is not enlarged and tall [14]. The protocone of the P4 is small, rounded and directed mostly lingually, as in *D. avus*. This pattern differs from the typical pattern observed in *L. culpaeus*, which has a more reduced protocone that is more mesially displaced, and in *D. australis*, where it is more reduced [5,7].

The p4 exhibits an acute distal cingulum as in *Dusicyon*, differing from the wide and straight distal border found in *L. culpaeus* [5,7] but lacks a second distal accessory cusp. The presence of a second distal accessory cusp is usually observed in *D. avus* but is absent in several specimens [5,7,8]. The hypoconulid of the m1 appears to be reduced to a very low cingulum. However, the talonid is partially worn and lacks enamel on its distolabial extreme, complicating this character’s interpretation. *Dusicyon avus* usually bears a well-elevated hypoconulid (e.g. [5,7,8]), but in some specimens, this structure is reduced or absent [7]. On the other hand, *D. australis* has a more reduced protoconid in the m1, and the principal cusps of the premolars are taller and more acute [5,7,78].

**Figure 3.**
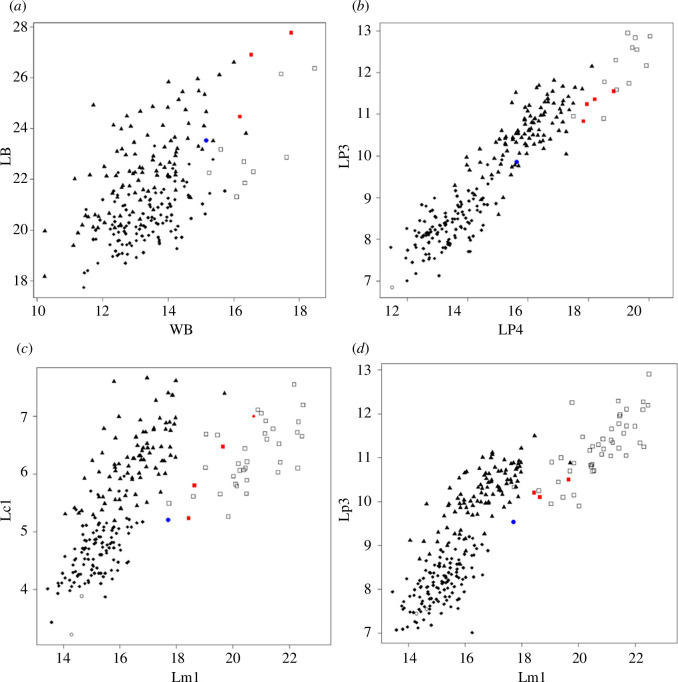
Bivariate graphs of craniodental measurements. Blue circle: specimen CS/91; white square: *Dusicyon avus*; red square: *D. australis*; black triangle: *Lycalopex culpaeus*; black rhombus: *L. gymnocercus*. (*a*) Length (LB) versus width (WB) of the bulla (mm). (*b*) Length of the fourth upper premolar (LP4) versus the length of the third upper premolar (LP3; mm). (*c*) Length of the first lower canine (Lc1) versus the length of the first lower molar (Lm1; mm). (*d*) Length of the third lower premolar (Lp3) versus the length of the first lower molar (Lm1; mm).

Less than half of the craniodental measurements of the CS/91 (e.g. the length of the bulla, the height of the mandible ramus, p2 and m2) lies below the known metric range of *D. avus*, CS/91 being smaller, but the difference is not very large (the median difference of the 14 smaller measurements is 0.6 mm; and the largest one that corresponds to the bulla length is 2.23 mm; electronic supplementary material, table S2; figure 3*a*). On the other hand, the measurements are within the range of *L. culpaeus*, indicating that overall size is congruent with this species (electronic supplementary material, table S2), something that could be observed in the biplot of several measurements (e.g. L/W of P3, C1, M1, LM2 versus LM1; figure 3; electronic supplementary material, figure S4) and in some PCA analyses (e.g. PCA based on P4–M2 measurements; electronic supplementary material, figure S5*a*).

Nevertheless, these analyses also show that the CS/91 specimen differs in the relative size of some tooth measurements from modern *L. culpaeus* populations, indicating that the m1 is proportionally longer than the length of the p3, p4, m2 and the width of the c1 (figure 3*c*; electronic supplementary material, figure S4*j*–*l*). CS/91 is more similar in this proportion to *Dusicyon*, which was previously described as having a proportional longer lower carnassial [5,7,8,79]. The PCA based on the height of the mandible and measurements of the c1–m2 placed the CS/91 in an intermediated position between *L. culpaeus*, *L. gymnocercus* and *Dusicyon* but closer to the latter (electronic supplementary material, figure S5*a*,*b*).

As expected, the CVs of *D. avus* increase when the specimen CS/91 is included, but the increase is small (with a median increase of 0.348 and a maximum, for the length of the P2, of 3.82; electronic supplementary material, table S2), and the CVs do not lie outside that observed in modern canids [54]. Compared with the sample of modern specimens of *L. culpaeus*, only 11 variables of 44 of the *D. avus* + CS/91 sample have larger CVs. However, their variation is small and non-significant (electronic supplementary material, table S2). Only the Lm2 has a significant difference in variance using the Cope and Lacy test but not with the Fligner–Killeen test. When CS/91 is excluded from the sample of *D. avus*, the Cope and Lacy test is also significant, indicating that the difference with the modern species is not solely caused by the inclusion of our archaeological specimen.

In summary, the combined morphological evidence suggests that CS/91 belongs to *D. avus* and that the observed differences with this species could be explained by its intraspecific variation.

### 3.2. Genetic analyses

DNA preservation was extremely poor, with only 0.08% mitochondrial endogenous DNA content. Mapping our data against the modern dog mitochondrial DNA reference genome yielded a higher depth of coverage when compared to that of the maned wolf *Ch. brachyurus* or the sechuran fox *L. sechurae* (electronic supplementary material, table S6). Unfortunately, the low number of reads prevented us from conducting an authentication of our generated data through mapDamage2 v.2.0.8 [80] as the threshold required for a minimum number of reads was not reached. For the phylogenetic analyses, we used consensus sequences mapped to *Ca. familiaris*. Specifically, we focused on a 684 bp fragment of the COII and a 1141 bp fragment of cytb owing to the available canid South American sequence on GenBank.

**Figure 4.**
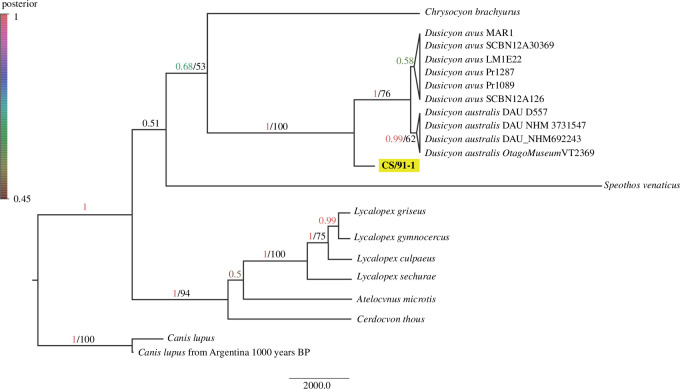
Phylogenetic relationship of South American canids. The phylogenetic tree illustrates the placement of the CS/91-1 sample within the *Dusicyon* genus. The tree is based on analysing the concatenated 1825 bp of mitochondrial COII and cytb genes. Nodal support values indicate the Bayesian posterior probabilities/maximum likelihood bootstrap values, with values above 0.5/50 displayed. The CS/91-1 sample is highlighted in yellow.

The ML and Bayesian phylogenetic reconstructions based on our data and 11 South American canid species produced identical highly resolved tree topology, both placing our sample unequivocally within the *Dusicyon* clade with a strong BS support of 100 and a posterior probability of 1 (figure 4). These results indicate that our CS/91-1 sample does not belong to the *Canis*, *Speothos* or *Chrysocyon* genera. Instead, it falls within the *Dusicyon* genus.

### 3.3. *Dusicyon* hybridization inference

Using the cytb divergence proxy, we predicted the sterility of hybrids between *Canis* and both *Dusicyon* species. The genetic divergences between *Ca. familiaris* and *D. avus* or *Ca. familiaris* and *D. australis* were determined to be 17.8% and 14.5%, respectively. These values fall into category 2, according to [70], indicating the potential for producing infertile hybrid offspring.

There are two categories along the spectrum of hybrid incompatibility. Category 1, from 0% to 7.2% cytb distance, is defined by mammalian species pairs capable of producing fertile F_1_ offspring of both sexes that can reproduce without backcrossing with a parent species. For example, *Canis latrans* (coyote) and *Canis lupus* (wolf) with a 5.4% distance value of cytb belong to category 1 [81–83].

Category 2, greater than 8% distance value of cytb, is defined by pairs of species that can produce viable F_1_ offspring but follow Haldane’s rule, and thus only female F_1_ can reproduce by backcrossing with a parent species. Category 2 also includes species pairs that can produce live offspring, but the F_1_s are infertile. For example, *Sus domesticus* (domestic pig) and *Babyrousa babyrussa* (babirusa) with a 12.9% distance [84].

On the other hand, the divergence between the two *Dusicyon* species was found to be 3.4%, which falls into category 1 and suggests the possibility of fertile hybrid offspring.

### 3.4. Radiocarbon and stable isotope analyses

**Figure 5.**
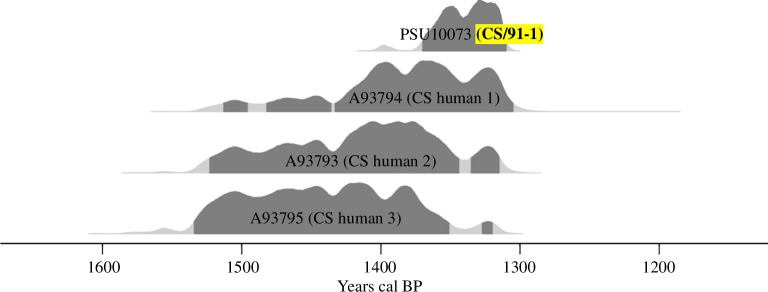
Radiocarbon dating of canid bone CS/91-1 shows contemporaneity with three previously dated human bones (1520 ± 13 yr ^14^C BP) [49].

Radiocarbon dating of the CS/91-1 bone provided an age of 1505 ± 15 yr ^14^C BP (Penn State University (PSU)-10073, 2σ: 1370-1310 cal BP; electronic supplementary material, table S9). This age is contemporary with the dates obtained from human bones found at the Cañada Seca site, which have an average age of 1520 ± 13 yr ^14^C BP (figure 5).

**Figure 6.**
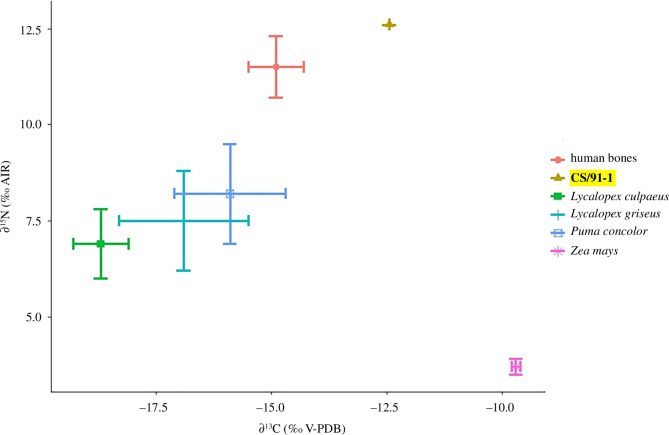
Carbon and nitrogen stable isotope values (δ^13^C_col_ and δ^15^N) comparison among wild carnivores (*Puma concolor, Lycalopex culpaeus* and *Lycalopex griseus*), human bones and CS/91-1 canid specimen.

Carbon and nitrogen stable isotopes of the CS/91-1 bone yield a δ^13^C_col_ value of −12.4‰ and δ^15^N value of 12.6‰ (laboratory code MSR-651), with a C : N atomic ratio of 3.18 (electronic supplementary material, table S8). The isotopic values obtained from CS/91-1 are significantly different from those of other wild carnivores in the study region, with an average enrichment of approximately 4.7‰ in δ^13^C_col_ and 5‰ in δ^15^N (figure 6). These findings indicate that the diet of the CS/91 canid varied from that of other wild carnivores in the region, pointing to a higher intake of C4 resources (probably maize) and a higher distinct trophic level. Furthermore, the isotopic results of CS/91-1 are more similar to the values obtained from adult humans found in the same archaeological context ([49]; figure 6). Specifically, the CS/91-1 result is enriched by 2.5‰ in δ^13^C_col_ and 1.1‰ in δ^15^N compared with the average values of the humans.

## 4. Discussion

### 4.1. An extinct fox outside of its known geographical distribution

Characterizing the CS/91 fox specimen from the Cañada Seca burial site provides valuable insights into the distribution of extinct canids and their interactions with human societies. Through a combination of morphological and genetic analyses, we confirmed its classification within the extinct genus *Dusicyon*, specifically *D. avus*. The well-preserved cranial and dental remains support this taxonomic assignment, highlighting the robust morphological characteristics observed in the specimen.

The genetic analysis using ancient DNA, although of poor quality, also provides additional support and firmly places the specimen within the *Dusicyon* clade. The examined sample does not group with *D. australis* or *D. avus* on the tree. This can be attributed to the constrained size of the sequenced cytb fragment available for the *Dusicyon avus* clade. Moreover, the precise segment of this compact fragment that exhibits synapomorphies for *Dusicyon avus* presents missing data in the sample analysed.

**Figure 7.**
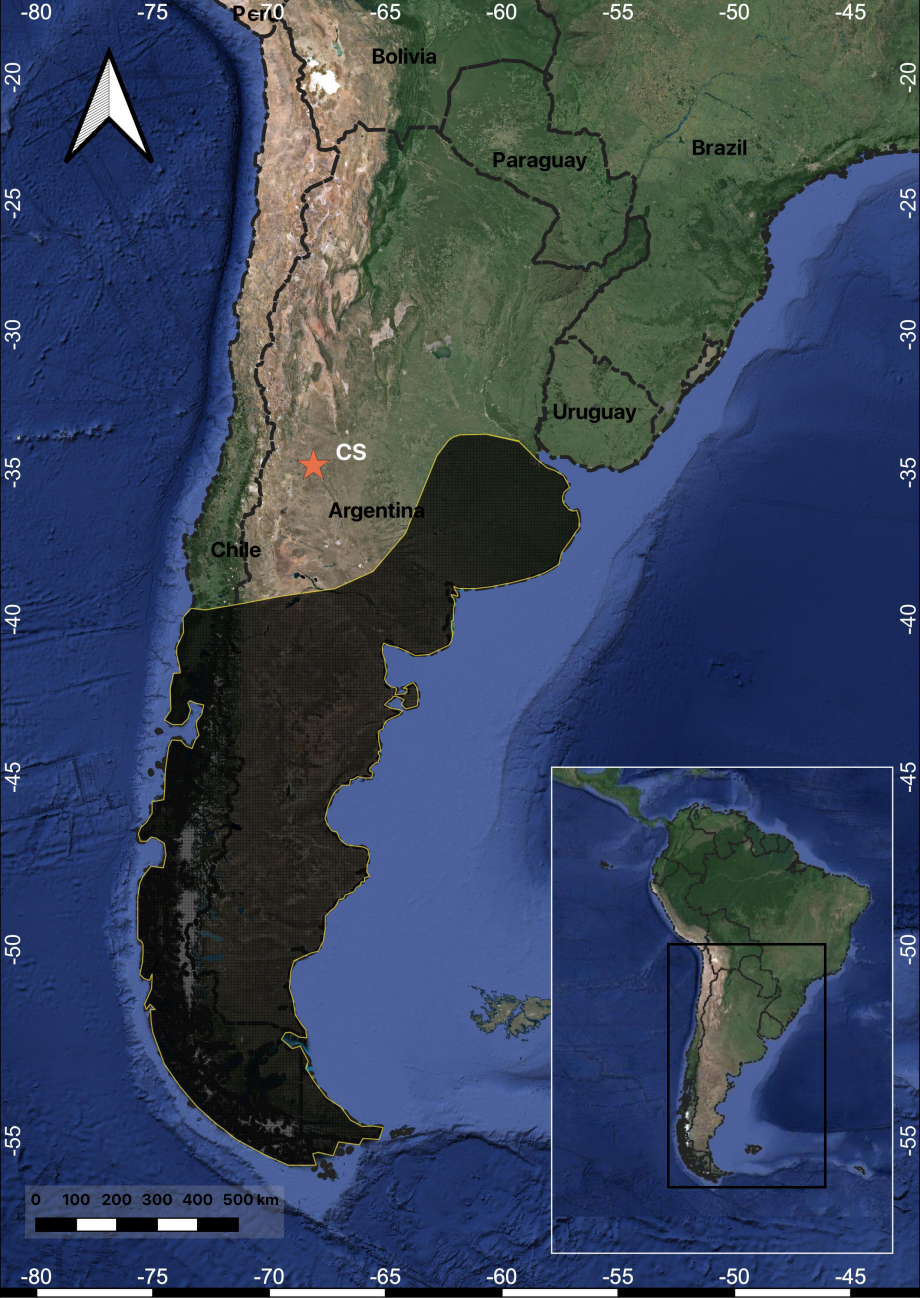
Distribution of *D. avus* and location of the CS/91 site in the Southern Cone of South America during the Holocene. This map illustrates the previous distribution of *D. avus*, represented by shading. The red star indicates the location of the CS site, where the CS/91 fox specimen was discovered. Note the presence of the CS/91 specimen outside the previously known distribution range of *D. avus*.

The CS/91 fox specimen falls outside its previously documented geographical distribution (figure 7) and extends the known range of the *Dusicyon* genus several hundred kilometres to the west and north [5]. The low mobility inferred by δ^18^O isotope from Cañada Seca humans implies this wild canid must have once been present in the immediate vicinity [49]. The southern Mendoza province is congruent with the environmental conditions similar to those of Patagonia [85], and some simulated distribution models generated for this fox included the region of the findings as a potential habitat for the species [5].

### 4.2. Reproductive compatibility

We wanted to address one of the possible causes of the extinction of *Dusicyon*, namely hybridization with domestic dogs introduced in the region [5,8,86]. The hypothesis is that hybridization between *D. avus* and domestic dogs could have led to the proliferation of hybrids in the population. These hybrids interbreed with each other and with individuals of both parent species. With each successive generation, the genetic influence of domestic dogs on the hybrid population would increase. This process could eventually lead to the disappearance of the alleles from *D. avus*. As a result, the *D. avus* species would gradually be lost in the hybrid genetic pool, which could have led to its extinction.

Our genetic divergence analysis suggests that this is unlikely. The genetic divergence values between *Canis* and *Dusicyon* species fall into category 2, suggesting a low probability of producing viable and fertile hybrid offspring. Therefore, the hybridization would not have resulted in a proliferation of hybrids capable of competing with the parent species or contributing to their extinction.

Although hybridization may have had some influence on population dynamics, by not producing fertile offspring, its contribution to the extinction event is limited. Our findings challenge previous hypotheses [8,86]. It seems more likely that other factors, such as anthropogenic effects and environmental changes, contributed to the extinction of *D. avus* populations.

### 4.3. Human*–Dusicyon* relationships

Although the loss of contextual information has made it difficult to ascertain the exact spatial association between this CS/91-1 fox and the human bones, direct radiocarbon date implies that the *D. avus* individual was part of the same human burial context. It is important to note that the CS site comprises commingled skeletal remains, and it is impossible to determine the nature of the associations between human skeletons and fox elements. Nevertheless, it is possible that the fox was intentionally inhumated in this burial context, becoming the first record of a complete skeleton of this fox species buried alongside humans.

The co-burial of humans and foxes is a rare archaeological record worldwide (e.g. [27,36]) and suggests a cultural or symbolic significance. Although the reasons for its inclusion in a mortuary context remain unclear, the most plausible explanation is that this fox was a valuable companion to the hunter-gatherer groups. Its strong bond with human individuals during its life would have been the primary factor for its placement as a grave good after the death of its owners or the people with whom it interacted.

The stable isotope analysis of carbon and nitrogen further supports this interpretation, revealing a similar dietary pattern between CS/91 and the human individuals found in the same archaeological context rather than a typical carnivorous diet. In other words, the hunter-gatherers fully incorporated this wild animal into their ecological and cultural niche, possibly through systematic feeding.

## 5. Conclusions

The CS/91 specimen provides valuable insights into extinct foxes' distribution, characteristics and ecological interactions. The combined morphological and genetic data analysis supports its classification as a *D. avus* canid with unique size characteristics, indicating that there were individuals of *D. avus* smaller than previously known. The presence of this fox outside its known distribution range expands our understanding of its geographical extent. The close association with human remains and shared dietary patterns suggests that this was a valuable individual, maybe even a companion or a pet for the hunter-gatherers during the late Holocene. Moreover, this record of a co-burial extends previously recognized symbolic interactions between hunter-gatherers and *D. avus* during the Holocene. The evidence indicates that some specimens of this extinct fox were in synanthropy during the late Holocene.

In terms of interactions between *D. avus* and domestic dogs, if they interbred or mixed, the absence of viable and fertile hybrid offspring suggests that this process was not a determining factor in the extinction of *D. avus* populations. While hybridization may have influenced population dynamics to some extent, its contribution to the extinction event appears to be limited by the lack of fertile offspring.

## Data Availability

Genetic Database: The public Sequence Read Archive (SRA) submission for CS/91-1 is PRJNA1001383 [86]. Electronic supplementary material is available online [87].
